# Novel humanized anti-CD20 antibody BM-ca binds to a unique epitope and exerts stronger cellular activity than others

**DOI:** 10.1002/cam4.60

**Published:** 2013-02-20

**Authors:** Hideaki Kobayashi, Yuka Matsunaga, Yumiko Uchiyama, Kenji Nagura, Yasuhiko Komatsu

**Affiliations:** BioMedics Japan Inc5F, Tokyo-Suisan-Bldg, 4-18, Toyomi-cho, Chuo-ku, Tokyo, 104-0055, Japan

**Keywords:** Anti-CD20 antibody, cancer chemotherapeutics, epitope, lymphoma, ofatumumab, rituximab

## Abstract

Cellular activity of BM-ca, a novel humanized anti-CD20 antibody, was quantitatively compared with that of two other anti-CD20 antibodies used for clinical practice, rituximab and ofatumumab. The results of a complement-dependent cytotoxicity (CDC) assay revealed that the strongest antibody was ofatumumab, followed by BM-ca, with rituximab being the weakest. Ofatumumab and BM-ca were effective not only against rituximab-sensitive SU-DHL-4 cells but also against rituximab-resistant RC-K8 cells. In an antibody-dependent cell-mediated cytotoxicity (ADCC) assay, although the effective concentrations against SU-DHL-4 cells were almost the same among these three antibodies, the maximum cytotoxic level was the highest for BM-ca. In an anti-cell proliferation assay using SU-DHL-4 cells, BM-ca was the most effective and ofatumumab, the weakest. Against RC-K8 cells, only BM-ca was effective. When combined with each of four cancer chemotherapeutics (prednisolone, vincristine, hydroxydaunorubicin, and cisplatin), BM-ca exerted the most effective combinatorial anti-cell proliferation activity. To assess the in vivo effect of BM-ca, we intravenously administered BM-ca into cynomolgus monkeys and found that the peripheral B-cell levels did not decrease in half of the animals. Sequencing of cDNA encoding CD20 of cynomolgus monkeys revealed that the responders and nonresponders had Leu/Pro (hetero) and Leu/Leu (homo) at amino acid (a.a.) position 160, respectively, suggesting that the epitope recognized by BM-ca was around this a.a. By analyzing reactivity to synthetic peptides, the epitope recognized by BM-ca was estimated to be a.a.'s 156–166, not shared with rituximab and ofatumumab. These results suggest BM-ca to be a promising anti-CD20 antibody having superior properties and recognizing a unique epitope.

## Introduction

CD20 is a promising target molecule of antibody drugs for treating a variety of B-cell-related diseases, including B-cell lymphoma, B-cell chronic lymphocytic leukemia, rheumatoid arthritis, multiple sclerosis, systemic lupus erythematosus, etc 1. Rituximab, which is the top runner of anti-CD20 antibody drugs, was launched in 1997 in the United States and is now used worldwide for a variety of indications 1,2. After the remarkable success of rituximab, many other attempts to develop other anti-CD20 antibodies were made 3,4. Over 15 years have passed since the debut of rituximab, but up to now only a single anti-CD20 antibody, ofatumumab, has been approved, and it for only a single indication, chronic lymphocytic leukemia 5. Many anti-CD20 antibodies under development have characteristics different from those of rituximab, for example, potentiated antibody-dependent cell-mediated cytotoxicity (ADCC) 6, stronger complement-dependent cytotoxicity (CDC) 7, no chimeric structure but rather a humanized or fully human structure, recognition of different epitopes, etc 1. However, such superiorities in vitro do not always reflect clinical efficacy. In other words, even now we do not sufficiently understand the key properties that allow these newer anti-CD20 antibodies to be more effective in vivo than the current champion, that is, rituximab.

BM-ca is a novel humanized anti-CD20 antibody having properties of both type-I and -II antibodies; that is, it has not only CDC but also direct cell death activities in addition to common ADCC activity 8. BM-ca is now in a clinical phase-I study in Japan. In previous studies, some comparisons had been made with other anti-CD20 antibodies, but most of these studies were qualitative rather than quantitative 8,9. In this study, we quantitatively compared the activity of BM-ca with that of the two approved anti-CD20 antibodies, rituximab and ofatumumab, in vitro, that is, their CDC, ADCC, and direct anti-cell proliferation activities. Furthermore, we compared their anti-cell proliferation activities in combination with cancer chemotherapeutic drugs. To know the molecular basis of the differences among these anti-CD20 antibodies, the epitope on CD20 recognized by BM-ca was extensively characterized. In the course of this study, we became aware of the molecular heterogeneity of CD20 in the cynomolgus monkey (*Macaca fascicularis*), which heterogeneity significantly affected the binding of BM-ca, but not that of rituximab, to this monkey's B cells.

## Materials and Methods

### Cells and cell culture

Two human B-cell lymphoma cell lines, RC-K8 and SU-DHL-4, were obtained from DSMZ (Deutsche Sammlung von Mikroorganismen und Zellkulturen GmbH, Braunschweig, Germany). Chinese hamster overy (CHO) cells constitutively expressing human CD20 molecules on their surface (CHO-CD20) were the same as those previously 10. These cell lines were cultured in RPMI-1640 (Roswell Park Memorial Institute-1640) medium supplemented with 10% fetal bovine serum (FBS; for RC-K8 and SU-DHL-4) or in CHO-S-SFM II medium (Invitrogen, Carlsbad, CA) supplemented with 0.4 mg/mL G418 (for CHO-CD20) at 37°C in a humidified chamber under a 5% CO_2_ atmosphere.

### Antibodies

BM-ca was produced by a recombinant CHO cell line and purified by a combination of several column chromatographies. Rituximab, ofatumumab, and infliximab, which are the ones used in clinical practice, were obtained from Hoffman-La Roche, GlaxoSmithKline, and Johnson & Johnson, respectively. Fluorescein isothiocyanate (FITC)-labeled anti-non-human primate CD20 (clone 2H7) was purchased from BD Biosciences (Franklin Lakes, NJ). FITC-labeled BM-ca and rituximab were synthesized by using a Fluorescein Labeling Kit-NH_2_ manufactured by DOJINDO (Kumamoto, Japan).

### Complement-dependent cytotoxicity

CDC activity was assayed by an AlamrBlue-based method that monitors the amount of viable cells. For this assay, 50,000 (SU-DHL-4) or 10,000 (RC-K8) cells in 50 μL of culture medium were seeded into each well of a 96-well black plate, into which 100 μL of premixed normal human serum complement (Quidel, San Diego, CA) and various concentrations of each antibody in culture medium were added. The final concentration of normal human serum complement was 1/16 of the original one. After the cells had been incubated at 37°C for 2 h, 15 μL of AlamarBlue (Invitrogen) was added, and then incubation was continued for another 16 h at 37°C in a humidified chamber under a 5% CO_2_ atmosphere. Viable cells in each well were monitored by measuring fluorescence at 590 nm (with excitation at 535 nm).

### Antibody-dependent cell-mediated cytotoxicity

ADCC activity was assayed by using a method to monitor dead cells by incubating cells with Calcein-AM, which labels viable cells. As the effector cells for the ADCC assay, peripheral blood mononuclear cells (PBMC) were prepared from three healthy volunteers after having obtained informed consent. In this assay, 20,000 (SU-DHL-4) or 40,000 (RC-K8) cells were seeded into each well of a 96-well U-bottom plate after the cells had been prelabeled with Calcein-AM by incubating them with 10 μmol/L Calcein-AM for 30 min. Then various concentrations of antibodies and effector cells (E:T ratio = 30:1) were added and mixed to elicit ADCC. After incubation of the cells for 3 h at 37°C in a 5% CO_2_ humidified incubator, the fluorescence of the culture supernatant was measured at 528 nm (with excitation at 480 nm).

### Anti-cell proliferation activity

The direct effect of each antibody on cell proliferation of B-lymphoma cells was studied by using AlamarBlue. For this test, 2500 cells in 50 μL of culture medium were seeded into each well of a 96-well black plate and incubated for 24 h at 37°C in a humidified chamber under a 5% CO_2_ atmosphere. Next, various concentrations of antibodies (50 μL) were added to each well, and the cultures were incubated for 3 days. After the addition of 10 μL of AlamarBlue, the cells were then incubated for another 4 h, after which the fluorescence at 590 nm (with excitation at 530 nm) was monitored.

### In vivo test in cynomolgus monkey

Although a repeated dose (i.v. once a week × four times) toxicity test using BM-ca was performed in cynomolgus monkeys, only the results about B-cell levels in circulation are described in this report. Six (three males and three females), six (three males and three females), and 10 (five males and five females) cynomolgus monkeys purchased from Japan Laboratory Animals, Inc. (Tokyo, Japan) were administered i.v. with BM-ca at a dose of 10, 20, and 50 mg/kg, respectively, once a week × four times (days 1, 8, 15, and 22). Before the first administration, and at day-8 (before administration), -9, -15 (before administration), -16, and -23 for all animals, and additionally at day-10 and -29 for four animals of the 50 mg/kg group, blood was withdrawn, and the percentage of CD20-positive cells in the lymphocyte population was determined by flow cytometry with aFACSCalibur-4S (BD Biosciences) after the cells had been stained with FITC-labeled anti-non-human primate CD20 (for method see below).

### Flow cytometry

Reactivity of anti-CD20 antibody against monkey's peripheral lymphocytes was determined by flow cytometry after the cells had been stained with FITC-labeled anti-CD20 antibodies. For analyzing B-cell levels after BM-ca administration, FITC-labeled anti-non-human primate CD20 was used. For comparing reactivity of BM-ca and rituximab against monkey's B cells, FITC-labeled BM-ca and rituximab were used. For this comparison study, not only cynomolgus monkeys (*Macaca fascicularis*) but also rhesus monkeys (*Macaca mulatta*) were used. Whole blood withdrawn from healthy monkeys was firstly stained with FITC-labeled anti-CD20 antibodies, and red blood cells were lysed with BD FACS lysing solution. After the residual cells had been washed with phosphate buffered saline (PBS) containing 4% FBS, the samples were subjected to flow cytometry. In most of the studies, we employed the FACSCalibur-4S described above; but in some studies a Guava (Millipore, Billerica, MA) was used as the flow cytometer.

### DNA sequence analysis of cynomolgus monkey's CD20 molecule

Primers were designed based on the predicted cDNA sequence of rhesus monkey CD20 (RefSeq: XM_001086364.1), since no corresponding sequence for cynomolgus monkeys had been available when this study was performed. Forward and reverse primers for cDNA cloning were TP81 (ATG ACA ACA CCC AGA AAT TCA GTA AA) and TP82 (TTA AGG AGA GCT GTC ATT TTC TAT TG), respectively, and the forward and reverse primers for sequencing were TP83 (ATG ACA ACA CCC AGA AAT TCA) and TP84 (TTA AGG AGA GCT GTC ATT TTC), respectively. In this study, since the primary purpose of sequencing CD20 was to assess the heterogeneity in both small and large extracellular loops, primers for DNA sequencing were not necessarily set outside of the open reading frame (ORF). After total RNA had been purified from whole blood by using Ribo-Pure Blood (Applied Biosystems, Foster City, CA), cDNAs encoding CD20 were amplified by RT-PCR (reverse transcription polymerase chain reaction) with primers TP81 and TP82. Their DNA sequences were determined at Macrogen Japan (Tokyo, Japan) with primers TP83 and TP84.

### Enzyme-linked immunosorbent assay

For examination of the ability of the anti-CD20 antibodies to bind to the potential epitope of BM-ca, 10 different 27-mer peptides corresponding to amino acid (a.a.) 140–166 with single a.a. replacement from human sequence were synthesized at Sigma-Aldrich (Hokkaido, Japan). At their N-termini, a biotin residue was added to anchor the peptides to the surface of NeutrAvidin-coated enzyme-linked immunosorbent assay (ELISA) plates (Reacti-Bind NeutrAvidine Bindings High Binding Capacity Coated 96-well Plate, Thermo, Waltham, MA). The peptides synthesized were CD20_140-166H (NIKIS HFLKM ESLNF IRAHT PYINI YN), CD20_140-166H_F154L (NIKIS HFLKM ESLN**L** IRAHT PYINI YN), CD20_140-166H_R156Q (NIKIS HFLKM ESLNF I**Q**AHT PYINI YN), CD20_140-166H_A157V (NIKIS HFLKM ESLNF IR**V**HT PYINI YN), CD20_140-166H_H158S (NIKIS HFLKM ESLNF IRA**S**T PYINI YN), CD20_140-166H_T159K (NIKIS HFLKM ESLNF IRAH**K** PYINI YN), CD20_140-166H_P160L (NIKIS HFLKM ESLNF IRAHT **L**YINI YN), CD20_140-166H_Y162V (NIKIS HFLKM ESLNF IRAHT P**V**INI YN), CD20_140-166H_N163D (NIKIS HFLKM ESLNF IRAHT PYI**D**I YN), and CD20_140-166H_N166D (NIKIS HFLKM ESLNF IRAHT PYINI Y**D**). The NetrAvidin-coated plates were incubated at 4°C overnight with a 1 μmol/L concentration of each peptide in PBS containing 0.1% bovine serum albumin (BSA) and 0.05% Tween20 (BSA-PBST), washed five times with PBS containing 0.05% Tween 20 (PBST), and blocked with 1% casein in PBST at room temperature for 1 h. After having been washed five times with PBST, the plates were incubated at room temperature for 1 h with various concentrations of antibodies in PBST under shaking. After another wash with PBST as above, the bound antibodies were detected with horseradish peroxidase (HRP)-labeled anti-human IgG (Bethyl, Montgomery, TX) and 3,3′,5,5′-tetramethylbenzidine (TMB) as a reporter dye (absorbance at 540 nm). As a positive control experiment, CD20-expressing CHO cells were coated on an ELISA plate and used as the natural antigen 10.

### Animal study

All animal studies reported here were performed with the approval of the institutional animal use and care committee of CMIC Bioresearch Center Co., Ltd. (Yamanashi, Japan).

## Results

### CDC activities of anti-CD20 antibodies

**Figure 1:**
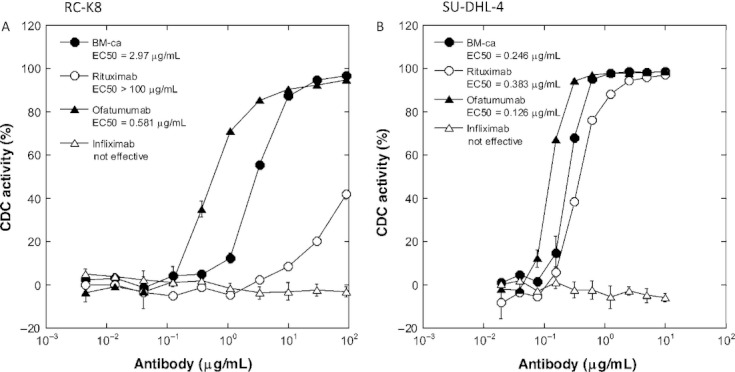
CDC activity of BM-ca (•), rituximab (○), ofatumumab (▴), and infliximab (▵) in RC-K8 (A) and SU-DHL-4 (B) cells. In all, 10,000 (RC-K8) and 50,000 (SU-DHL-4) cells were mixed with various concentration of antibodies and normal human serum complement (final dilution = 1/16) and incubated at 37°C for 2 h. Viable cells were fluorometrically monitored by using AlamarBlue, as described in Materials and Methods. CDC activity was calculated according to the following formula: [CDC activity (%)] = (1 – ([Fluorescence with antibody] − [Fluorescence without cells and antibody])/([Fluorescence without antibody] − [Fluorescence without cells and antibody])) × 100. In the figures, 50% effective concentration (EC50) of each antibody was graphically determined and depicted. Each point represent the mean ± SD (*n* = 2).

The results of dose-dependent comparison of CDC activities among anti-CD20 antibodies are shown in Figure 1 for both rituximab-resistant RC-K8 (Fig. 1A) and -sensitive SU-DHL-4 (Fig. 1B) cells 11. As shown in the figures, of atumumab had the strongest anti-CD20 antibody activity in the CDC assay, followed by BM-ca; and rituximab displayed the weakest one. Especially in RC-K8 cells, rituximab only exerted very weak CDC activity (Fig. 1A).

### ADCC activities of anti-CD20 antibodies

**Figure 2:**
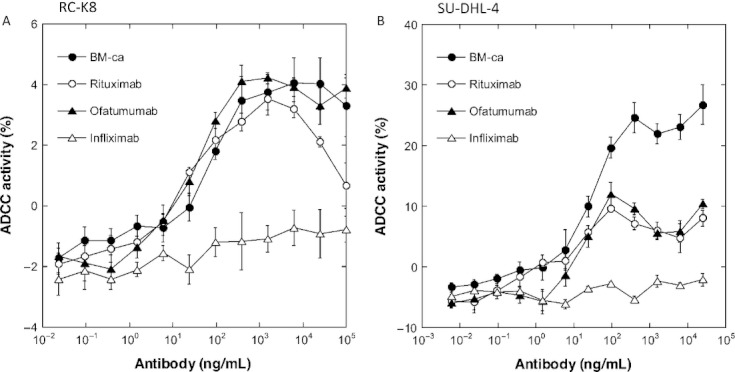
ADCC activity of BM-ca (•), rituximab (○), ofatumumab (▴), and infliximab (▵) in RC-K8 (A) and SU-DHL-4 (B) cells. RC-K8 (40,000) and SU-DHL-4 (20,000) cells prelabeled with Calcein were mixed with various concentrations of antibodies and PBMC (E:T ratio = 30:1) and incubated at 37°C for 3 h. The cells were then lysed and fluorometrically monitored by measuring the fluorescence of the supernatant, as described in Materials and Methods. ADCC activity was calculated according to the following formula: [ADCC activity (%)] = ([Fluorescence with antibody] − [Fluorescence without antibody])/([Maximum Fluorescence] − [Fluorescence without antibody]) × 100, where Maximum Fluorescence is the value after treatment of target cells with 1% Triton X-100. Each point represents the mean ± SD (*n* = 9 for BM-ca; *n* = 3 for other antibodies).

Dose-dependent comparison of ADCC activities among the three anti-CD20 antibodies gave the results shown in Figure 2. Although there existed no significant differences in ADCC activities in RC-K8 cells, in SU-DHL-4 cells BM-ca lysed a larger number of target cells (about 25%) than the two other antibodies (about 10%) above plateau concentrations. The effective concentration range was almost the same among these three antibodies.

### Anti-cell proliferation activity of anti-CD20 antibodies in solo and in combination with various cancer chemotherapeutics

**Figure 3:**
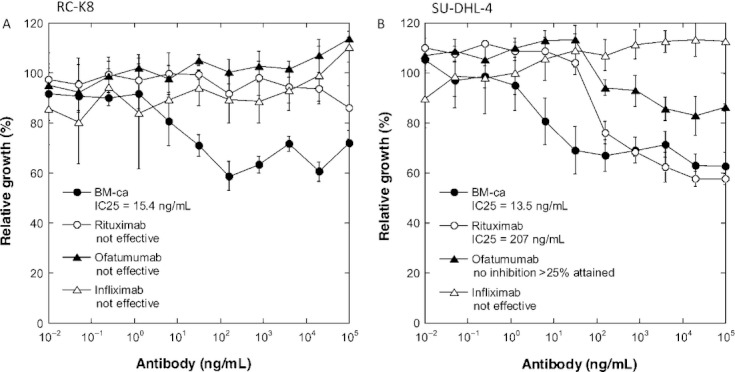
Anti-cell proliferation activity of BM-ca (•), rituximab (○), ofatumumab (▴), and infliximab (▵) in RC-K8 (A) and SU-DHL-4 (B) cells. The cells (2500) were seeded in each well of a 96-well plate, and various concentrations of antibodies were added the next day. After incubation at 37°C for 3 days in a humidified chamber under a 5% CO_2_ atmosphere, viable cells were fluorometrically monitored by using AlamarBlue, as described in Materials and Methods. Relative growth (%) values were calculated according to the following formula: [Relative growth (%)] = ([Fluorescence with antibody] − [Fluorescence without cells and antibody])/([Fluorescence without antibody] − [Fluorescence without cells and antibody]) × 100. The 25% inhibitory concentration (IC25) of each antibody was graphically determined and is depicted in the figures. Each point represents the mean ± SD (*n* = 4).

The findings on dose-dependent comparison of anti-cell proliferation activities of BM-ca, rituximab, ofatumumab, and infliximab are depicted in Figure 3. In accordance with previously reported results 12–15, no complete inhibition of cell proliferation by the anti-CD20 antibodies was observed in either RC-K8 or SU-DHL-4 cells. However, efficacies of these antibodies were largely different. Namely, in RC-K8 cells, neither rituximab nor ofatumumab was effective. Only BM-ca was effective, where its 25% inhibition concentration (IC25) was 15.4 ng/mL. In SU-DHL-4 cells, although all three anti-CD20 antibodies inhibited cell proliferation to some degree, their effective concentrations were largely different. The strongest was BM-ca (IC25 = 13.5 ng/mL), followed by rituximab (IC25 = 207 ng/mL), whereas ofatumumab had only marginal activity. In both cell lines, the negative control antibody, infliximab, was ineffective.

**Figure 4:**
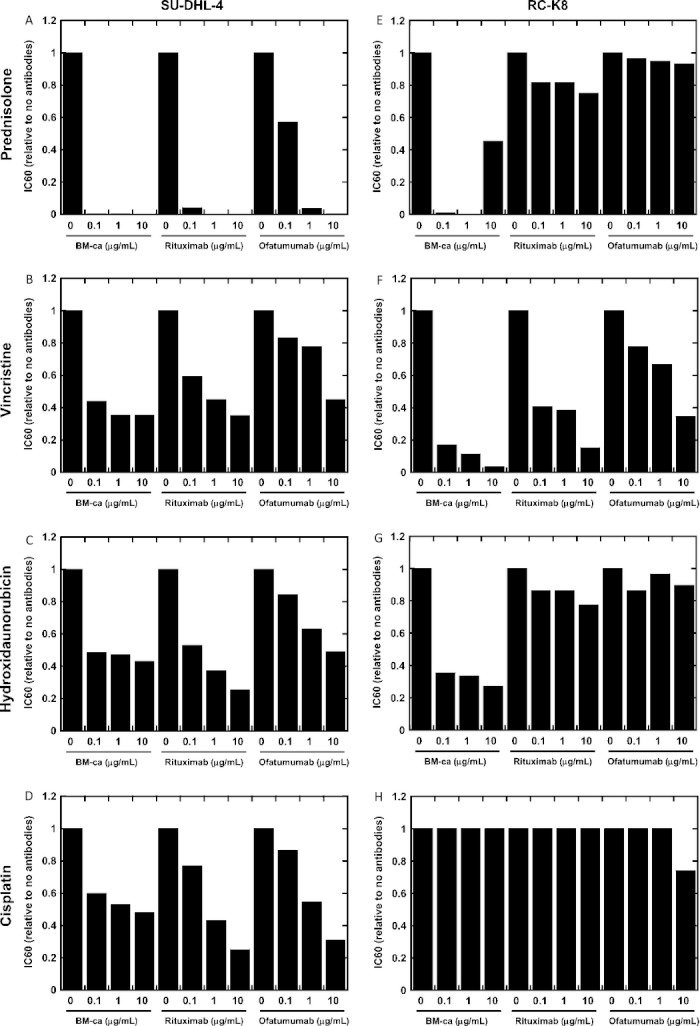
Effect of anti-CD20 antibodies on anti-cell proliferation activity of cancer chemotherapeutic drugs in SU-DHL-4 (A–D) and RC-K8 (E–H) cells. Various concentrations of prednisolone (A and E), vincristine (B and F), hydroxydaunorubicin (C and D), or cisplatin (D and H) were added to each well containing cells in combination with 0, 0.1, 1, or 10 μg/mL BM-ca, rituximab, or ofatumumab. Experimental condition was as the same as that described for Figure 3. Sixty percent inhibition concentration (IC60) of each cancer chemotherapeutic drug was determined from the dose-response curves (Figs. S1–S4), and the relative values to no-antibody-condition were determined. IC60s from which the relative values were calculated are summarized in Tables S1 and S2.

Next, we examined the effects of these anti-CD20 antibodies when combined with each of four cancer chemotherapeutic drugs, that is, prednisolone, vincristine, hydoxydaunorubicin, and cisplatin. In this study, we did not examine the effect of cyclophosphamide, although this drug is included in a popular CHOP (cyclophosphamide, hydroxydaunorubicin, oncovin, and prednisone or prednisolone) regimen with prednisolone, vincristine, and hydroxydaunorubicin, because in vivo activation is necessary for cyclophosphamide to become active 16. The results are summarized in Tables S1 and S2 for SU-DHL-4 and RC-K8 cells, respectively, and these tables show the change in the IC60 value of each of the cancer chemotherapeutics. The IC60 values relative to the value without antibodies are calculated and depicted in Figure 4 to show a whole image. All of the dose–response curves from which the IC60 values were derived are attached as supporting information (Figs. S1–S4). In this study, IC60 was used for comparing the combination effects instead of usual IC50, since in some conditions cell proliferation was inhibited >50% only with the antibody (without cancer chemotherapeutics), making overall comparison difficult. As shown in Figure 1 and Table S1, an increase in the concentration of any of the anti-CD20 antibodies decreased the IC60 values of all cancer chemotherapeutics in SU-DHL-4 cells, where BM-ca was the most effective at lower concentrations, followed by rituximab and then ofatumumab, indicating a promising combination effect in the clinical setting. In RC-K8 cells, only BM-ca significantly affected the IC60 value of prednisolone and hydroxydaunorubicin, and BM-ca was the most effective in combination with vincristine (Fig. 4 and Table S2), suggesting the superiority of BM-ca in combination therapy against rituximab-resistant tumors. However, all three anti-CD20 antibodies including BM-ca did not show combination effects with cisplatin in this cell line.

### B-cell depletion in cynomolgus monkeys after i.v. administration of BM-ca

**Figure 5:**
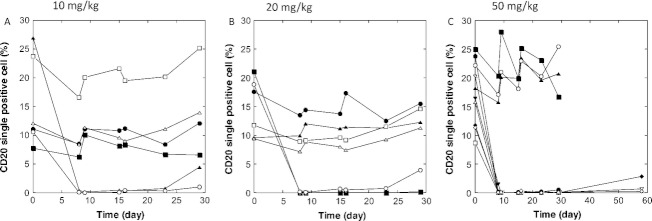
Change in percentage of CD20 single positive cells in the peripheral blood of cynomolgus monkeys treated with BM-ca. In all, 10 (A), 20 (B), or 50 (C) mg/kg of BM-ca was administered i.v. once a week × four times to 6, 6, and 10 cynomolgus monkeys, respectively. Peripheral blood was withdrawn at the indicated time points, and the percentage of CD20 single positive cells was determined by flow cytometry, as described in Materials and Methods. Different symbols indicate different individual animals. These data show that half of the animals did not respond to BM-ca.

In the course of our toxicity study using cynomolgus monkeys, we encountered a strange phenomenon regarding the efficacy of BM-ca, in vivo, that is, heterogeneity of the response to BM-ca (a decrease in circulating B-cell levels) after its administration i.v. (Fig. 5). Namely, four of six, four of six, and three of 10 (totally 11 of 22) animals did not respond to BM-ca administered i.v. at a dose of 10, 20, and 50 mg/kg, respectively. This kind of study using cynomolgus monkeys was reported earlier regarding the effects of both rituximab 17 and ofatumumab 18; but such a heterogeneity of response to these anti-CD20 antibodies was not reported 17,18, thus suggesting this heterogeneity to be related to some uniqueness of BM-ca.

### Flow cytometric analysis of monkey's B-cell reactivity with BM-ca and rituximab

To confirm that nonresponders to BM-ca were specific to cynomolgus monkeys, we compared the reactivity of FITC-labeled BM-ca and rituximab to peripheral lymphocytes of cynomolgus monkeys and rhesus monkeys. The B-cell ratio was determined by flow cytometry after gating the lymphocyte population based on the front and side scatterings (Fig. S5). The resulting histograms are shown in Figure 1, and the calculated percentages of B cells are summarized in Table 1 (see column for FACS Calibur). The percentages of B cells in the population of peripheral lymphocytes obtained from all six rhesus monkeys (No. 1–6) were above several percent for both BM-ca and rituximab, indicating no heterogeneity in reactivity toward either BM-ca or rituximab. On the contrary, although peripheral lymphocytes obtained from 6 cynomolgus monkeys (No. 7–12) bound to rituximab, those from three of them (No. 8, 10, and 12) did not bind BM-ca. Five of them (No. 7, 8, 9, 11, and 12) and one other cynomolgus monkey (No. 13) were analyzed with the other instrument (Guava, Millipore), and the resultant data confirmed the existence of two populations of cynomolgus monkeys having different binding properties with respect to BM-ca (see column for Guava). The apparent difference in the percentage values between FACS Calibur and Guava is thought to be caused by the difference in the instruments, and the difference in the percentage values in positive individuals between BM-ca and rituximab is thought to be derived from the difference in the quality of FITC-labeled antibodies.

**Table 1:** Flow cytometric analysis of B cells in monkey's blood

Animal no.	Species	Sex	Test compound	Positive%^1^ (FACS Calibur)	Positive%^2^ (Guava)	Reactivity^3^	160th A.A.^4^
1	*Macaca mulatta*	F	–	0.00	N.T.	−	N.T.
			BM-ca	5.08		+
			Rituximab	11.04	+	
2	*M. mulatta*	F	–	0.00	N.T.	−	N.T.
			BM-ca	3.89		+
			Rituximab	8.60		+
3	*M. mulatta*	F	–	0.00	N.T.	−	N.T.
			BM-ca	3.49		+
			Rituximab	7.36		+
4	*M. mulatta*	F	–	0.00	N.T.	−	N.T.
			BM-ca	8.17		+
			Rituximab	10.95		+
5	*M. mulatta*	F	–	0.00	N.T.	−	N.T.
			BM-ca	4.12		+
			Rituximab	6.81		+
6	*M. mulatta*	F	–	0.00	N.T.	−	N.T.
			BM-ca	2.74		+
			Rituximab	5.56		+
7	*Macaca fascicularis*	M	–	0.00	N.T.	−	P/L
			BM-ca	2.64	12.80	+
			Rituximab	6.31	15.79	+
8	*M. fascicularis*	M	–	0.00	N.T.	−	L/L
			BM-ca	0.72	1.64	−
			Rituximab	11.08	15.07	+
9	*M. fascicularis*	M	–	0.00	N.T.	−	P/L
			BM-ca	8.77	27.58	+
			Rituximab	23.84	34.25	+
10	*M. fascicularis*	M	–	0.00	N.T.	−	N.T.
			BM-ca	0.65	N.T.	−
			Rituximab	4.66	N.T.	+
11	*M. fascicularis*	F	–	0.00	N.T.	−	P/L
			BM-ca	3.41	17.68	+
			Rituximab	9.20	19.77	+
12	*M. fascicularis*	F	–	0.00	N.T.	−	L/L
			BM-ca	0.52	9.00	−
			Rituximab	13.91	23.36	+
13	*M. fascicularis*	F	–	N.T.	N.T.	−	L/L
			BM-ca	N.T.	5.19	−
			Rituximab	N.T.	18.24	+

N.T., not tested.

1 B cell% was determined by the analysis using FACS calibur as a flow cytometer.

2 B cell% was determined by the analysis using guava as a flow cytometer.

3 Reactivity is categorized as positive (+), when B-cell% value is above 1% (FACS Calibur) and/or 10% (Guava).

4 Amino acid at position 160 of CD20 molecule determined by sequencing cDNAs encoding CD20 (see also Fig. 7).

**Figure 6:**
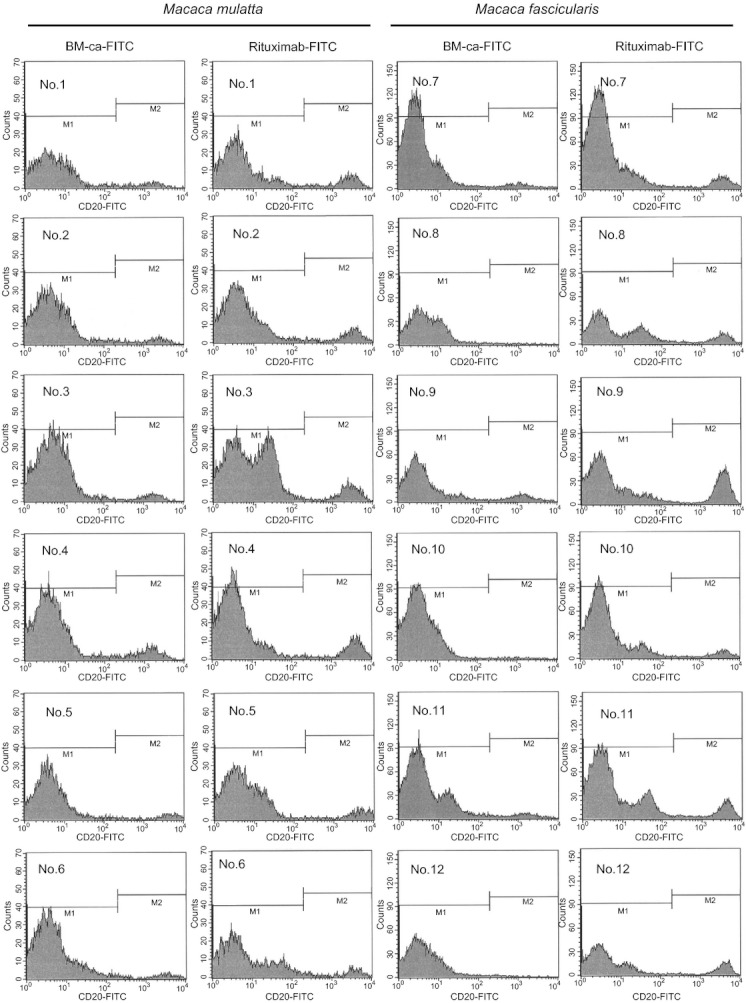
Flow cytometry analysis of reactivity to BM-ca of peripheral blood lymphocytes in rhesus (No. 1–6) and cynomolgus (No. 7–12) monkeys. Peripheral blood was withdrawn from rhesus (*Macaca mulatta*) and cynomolgus (*Macaca fascicularis*) monkeys, six of each; and the reactivity to FITC-labeled BM-ca and FITC-labeled rituximab was examined by use of flow cytometry. Histograms of the reactivity of lymphocytes to BM-ca (first and third columns) and rituximab (second and fourth columns) are depicted. All six rhesus monkeys were reactive with both BM-ca and rituximab. However, three of the six cynomolgus monkeys (No. 8, 10, and 12) did not react with BM-ca, whereas all six animals were reactive with rituximab.

### DNA sequence analysis of cynomolgus CD20 molecule

**Figure 7:**
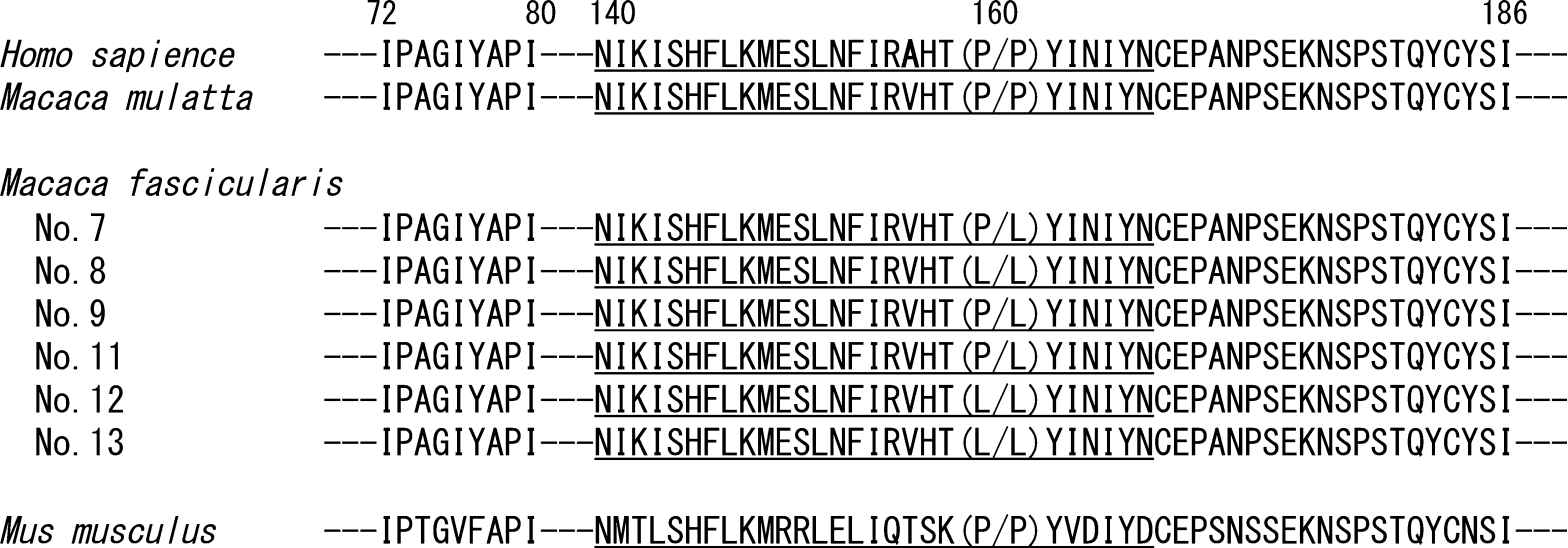
Amino acid sequences of extracellular loops in CD20 molecules. Sequences of *Homo sapiens* [make change in figure also], *Macaca mulatta*, and *Mus musculus* were from a database. Regarding *Macaca fascicularis*, sequences of six different animals were determined and are indicated. Positions 72–80 and 140–186 are small and large extracellular loops, respectively. Although the amino acid at position 160 is Pro in all 3 species other than *Macaca fascicularis*, Leu was found in addition to Pro in *Macaca fascilularis*. Peptides corresponding to the underlined position (140–166) were used in the epitope analysis described in Figure 8.

One probable cause of such a difference in reactivity toward BM-ca could be the existence of heterogeneity in the amino acid sequence at the site important for BM-ca binding. To know whether this was the case, we sequenced the cDNA encoding CD20 molecules expressed in six individual cynomolgus monkeys (No. 7, 8, 9, 11, 12, and 13). Full sequences of this cDNA are shown in Figure S6, and the deduced amino acid sequences of small and large extracellular loops are depicted in Figure 7 with the corresponding sequences of humans, rhesus monkey, and mice deposited in DNA databases. As clearly shown in Figure 7, two populations of animals having a different amino acid at position 160 existed. Namely, one population (No. 7, 9, and 10) was hetero at this site having both Leu and Pro, whereas another population (No. 8, 12, and 13) was homo at this site, having only Leu. Interestingly, these two populations exactly coincided with those of responder and nonresponder populations to BM-ca; that is, the Leu/Pro hetero population reacted with BM-ca, whereas the Leu/Leu homo population did not (Table 1).

### Epitope analysis of BM-ca with synthetic peptides

**Figure 8:**
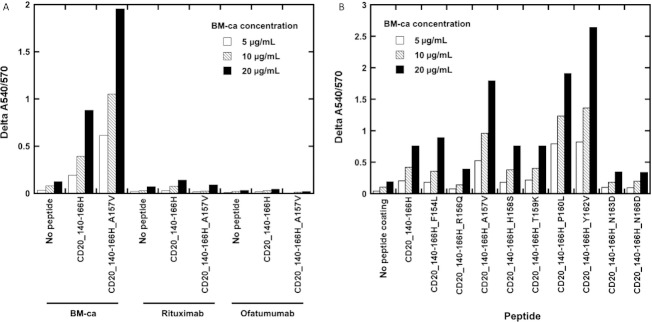
Epitope analysis of BM-ca by use of synthetic peptides. Peptides corresponding to position 140–166 of CD20 molecules with a variety of single amino acid replacement from the human sequence were synthesized and anchored to the wells of an ELISA plate via biotin residues introduced at their N-terminals. (A) Reactivity of BM-ca, rituximab, and ofatumumab to human (CD20_140-166H) and monkey (CD20_140-166H_A157V) sequences was examined at the concentrations of 5, 10, and 20 μg/mL, where only BM-ca strongly reacted with these peptides dose dependently. (B) Reactivity of BM-ca at various concentrations (5, 10, and 20 μg/mL) with various peptides having a different single point amino acid replacement was compared.

Since these results strongly suggest that the region in the large extracellular loop around position 160 was the epitope recognized by BM-ca, we synthesized peptides of a.a. 140–166 of human (CD20_140-166H) and monkey (CD20_140-166H_A157V) sequences having Pro at position 160 and examined their ability to bind to BM-ca, rituximab, and ofatumumab. As shown in Figure 8A, only BM-ca bound to these peptides dose dependently. Since all of these three anti-CD20 antibodies strongly bound to natural human CD20 antigen under the same assay conditions (Fig. S7), this result clearly indicates that this region was the epitope seen by BM-ca and was not shared with rituximab or ofatumumab. To know more details about the sites in CD20 important for binding to BM-ca, some amino acids (positions 154, 156, 158, 159, 162, 163, and 166), which are different from the corresponding amino acids of mouse CD20, were replaced by the mouse-type ones, since BM-ca does not bind to mouse CD20 (unpubl. results of ourselves). The results are described in Figure 8B. Among them, replacement at position 156, 163, or 166 resulted in decreased binding, whereas the replacement at 162 increased it. The change at position 154, 158, or 159 did not change the binding. We also examined the replacement at position 160 from Pro to Leu. Surprisingly, BM-ca bound to the peptide having Leu at position 160 instead of Pro stronger than to the original human sequence (Fig. 8B), in spite of the observation of a dramatic decrease in the binding of BM-ca to B cells in cynomolgus monkeys having Leu/Leu homo CD20 (Fig. 7 and Table 1). These results suggest that positions 156, 157, 162, 163, and 166 are especially important in the large extracellular loop of CD20 for binding to BM-ca.

## Discussion

In this study, we found that BM-ca was the strongest anti-CD20 antibody, compared with rituximab and ofatumumab, in ADCC and direct anti-cell proliferation assays. This is the first quantitative study showing the superiority of BM-ca in these two assays. On the contrary, in this study, the order of efficacy of these anti-CD20 antibodies in the CDC assay was ofatumumab > BM-ca > rituximab; although in the previous study, we had reported that BM-ca was stronger than ofatumumab when tested by this assay 9. This discrepancy might be attributable to the different quality of ofatumumab used in the previous and present studies, because in the previous study, we produced ofatumumab by ourselves by using a recombinant DNA technology based on the reported sequence of ofatumumab 9. In this study, however, we used ofatumumab that is utilized in the clinical situation, so it may be that the activity of ofatumumab in this study was stronger than that in the previous one.

In all of the cellular assays performed in this study, we used both SU-DHL-4 and RC-K8 as the target cells for comparison, and found that BM-ca was effective in RC-K8 cells as well as in SU-DHL-4 cells. Although it has been reported that RC-K8 cells are resistant to rituximab 11, the underlying mechanism by which the cells become resistant to rituximab has not yet been fully elucidated. Concerning the resistance of RC-K8 cells to rituximab in the CDC assay, one possibility is their higher expression of CD55 and/or CD59 19, which are cell surface inhibitors of complement 20. Also in our study, a relatively larger amount of complement was required for full CDC activity in RC-K8 cells. However, the observed resistance of RC-K8 cells in the direct cell proliferation inhibition assay might not be explained by the overexpression of CD55 and CD59. The fact that BM-ca could overcome the resistance to rituximab in RC-K8 cells in both CDC and anti-cell proliferation assays would suggest greater efficacy of BM-ca also in the clinical situation. On the contrary, because the resistance of RC-K8 cells to rituximab was not observed in the ADCC assay, the resistance mechanism might not be related to an ADCC-specific pathway. Since we used only two typical B-cell lymphoma cell lines in this study, same kind of quantitative comparison using other B-cell lymphoma and leukemia cell lines might be helpful to know the mechanisms underlying the differences in the activities among different anti-CD20 antibodies.

The anti-cell proliferation activity of anti-CD20 antibodies including BM-ca was mild in this study (maximum inhibition was 37–57% depending on the runs of experiments; see Fig. 3; Tables S1 and S2, and Figs. S1–S4), which is in consistent with the results of previous studies (maximum inhibition was about 30–70%) 12–15. Although some apoptotic activity of BM-ca has been reported 8, addition of BM-ca to the cell culture medium did not kill the cells for several days even if affecting the cell proliferation rate (data not shown), suggesting that induction of direct cell death (or apoptosis) might occur only in the conditions suffering from additional stresses, for example, low serum concentration, hypoxia, and coexistence of anti-cancer drugs.

Through three different quantitative cellular assays, we noticed differences among them in terms of the effective concentration of BM-ca. Namely, CDC required the highest concentration of BM-ca, whereas ADCC and anti-cell proliferation activity required only about a 1/10 to 1/100 lower concentration. These results suggest that larger fraction of CD20 molecules must be occupied by BM-ca for CDC activity than for the two other activities. In the clinical setting, this might mean that CDC activity would not endure after the blood concentration of BM-ca decreased, whereas ADCC and anti-cell proliferation activity would continue. Since BM-ca was effective at a lower concentration than rituximab or ofatumumab in terms of anti-cell proliferation activity, this effect of BM-ca would possibly continue longer after the blood concentration of anti-CD20 antibodies decreased. Furthermore, BM-ca was the most effective when combined with cancer chemotherapeutic drugs in the anti-cell proliferation assay, thus indicating this antibody to be promising for combination therapy as well. One dependable way to speculate the clinical superiority of BM-ca might be a study with mouse xenograft model inoculated with human lymphoma cell lines, for example, SU-DHL-4 and RC-K8. However, the results will not be straightforward because the effector cells and complement factors are not of human origin in mice. In fact superiority observed in xenograft model of anti-CD20 antibodies 6,21 has not led to the superiority in human. So we think that future clinical studies are necessary to know whether BM-ca is superior in human as well.

**Figure 9:**
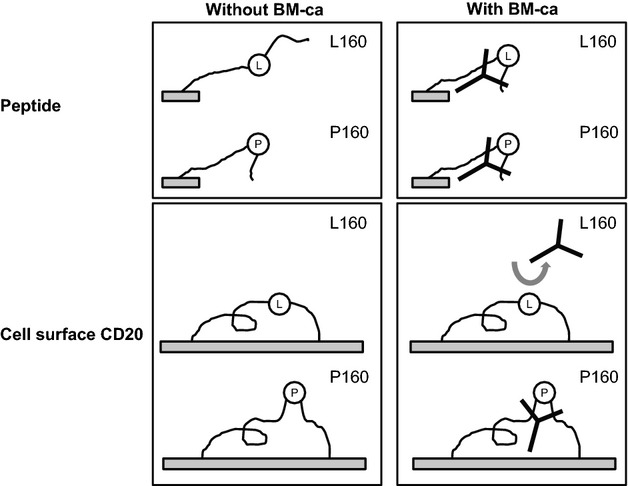
Schematic drawings of the interaction between BM-ca and its epitope. A hypothesis explaining the role of Pro160 is illustrated. In peptide, the amino acid chain can vend freely to fit the groove on BM-ca, even if Leu160 instead of Pro160. On the contrary, in cell surface CD20, Pro160 might be necessary to keep the vended structure in restricted circumstances of protein molecule.

Although the molecular basis for the differences in biological activities between BM-ca and the other anti-CD20 antibodies still remains unclear, one key factor would be the epitope on CD20. Pro160 was strongly suggested to play a critical role for BM-ca to bind to cell surface CD20 based on the result of DNA sequencing of cDNA encoding CD20 of cynomolgus monkeys, since the probability of accidental coincidence between no-binding of BM-ca and absence of Pro160 in six animals is thought to be very low (0.5^6^ = about 1.6%). We expect that this hypothesis will be confirmed by additional analysis of other cynomolgus monkeys. In addition to this, the peptide experiment suggested that the region of a.a. 156–166 (including Pro160) is the potential epitope of BM-ca not seen by rituximab or ofatumumab. However, the replacement of Pro160 with Leu in peptide did not reproduce the result of cellular experiment. We think that this apparent inconsistency between cellular and peptide experiments can be explained as follows. Namely, it can be thought that although peptides having Leu160 can freely vend to fit to the groove on BM-ca which recognizes CD20, Pro160 might be necessary to keep the vended structure in restricted circumstances of protein molecule (Fig. 9). More concrete conclusion about the epitope of BM-ca will be obtained by further dedicated experiments with systematic site-directed mutagenesis of cell surface CD20 molecules including Pro160, although such a full-scale recombinant study might be beyond the scope of this manuscript. These differences in the epitope might bring about differential activation of signaling pathways that trigger different biological activities.

The differential biological activity found in cynomolgus monkeys was a clue indicating the epitope of BM-ca to be around a.a. 160. The heterogeneity in amino acid at position 160 (Pro or Leu) in cynomolgus monkeys was found for the first time in this study. One sequence of cynomolgus CD20 is now present in the GenBank DNA database (EHH56190.1) 22, and it is the more abundant Leu160 type. Since Leu is not found in other monkeys, primates or humans at position 160 of CD20 (NCBI blastp search against EHH56190.1 on 3 August 2012), we cannot consider this change from an evolutional point of view for now. By extensively analyzing the sequence of CD20 molecules of various monkeys and primates, we should be able to obtain insight into the evolutional implication of this mutation.

## Supporting Information

**Figure S1.** Combination effects of anti-CD20 antibodies with prednisolone in the anti-cell proliferation assay. Effects of prednisolone at various concentrations were examined in the presence of 0 (•), 0.1 (○), 1 (▴), and 10 μg/mL (▵) of BM-ca (A and D), rituximab (B and E), or ofatumumab (C and F) in SU-DHL-4 (A–C) and RC-K8 (D–F) cells. Each point represents the mean ± SD (*n* = 4). **Figure S2.** Combination effects of anti-CD20 antibodies with vincristine in the anti-cell proliferation assay. Effects of vincristine at various concentrations were examined in the presence of 0 (•), 0.1 (○), 1 (▴), and 10 μg/mL (▵) of BM-ca (A and D), rituximab (B and E), or ofatumumab (C and F) in SU-DHL-4 (A–C) and RC-K8 (D–F) cells. Each point represents the mean ± SD (*n* = 4). **Figure S3.** Combination effects of anti-CD20 antibodies with hydroxydaunorubicin in the anti-cell proliferation assay. Effects of hydroxydaunorubicin at various concentrations were examined in the presence of 0 (•), 0.1 (○), 1 (▴), and 10 μg/mL (▵) of BM-ca (A and D), rituximab (B and E), or ofatumumab (C and F) in SU-DHL-4 (A–C) and RC-K8 (D–F) cells. Each point represents the mean ± SD (*n* = 4). **Figure S4.** Combination effects of anti-CD20 antibodies with cisplatin in the anti-cell proliferation assay. Effects of cisplatin at various concentrations were examined in the presence of 0 (•), 0.1 (○), 1 (▴), and 10 μg/mL (▵) of BM-ca (A and D), rituximab (B and E), or ofatumumab (C and F) in SU-DHL-4 (A–C) and RC-K8 (D–F) cells. Each point represents the mean ± SD (*n* = 4). **Figure S5.** Typical representations of gating of lymphocytes (A), histogram without antibody (B), that with BM-ca-FITC (C), and that with rituximab-FITC (D), in flow cytometry analysis of peripheral blood of monkeys (rhesus monkey; animal no. 1). M1 and M2 are negative and positive populations, respectively. **Figure S6.** Sequences of cDNAs encoding two different types of CD20 molecules in cynomolgus monkeys. In the a.a. 160 = Leu molecule, the nucleotide position 479 is T; whereas in the a.a. 160 = Pro molecule, it is C. **Figure S7.** Reactivity of BM-ca, rituximab, ofatumumab, and infliximab with CD20 molecule expressed on the surface of CHO cells in the ELISA assay under the same assay conditions as in Figure 8. **Table S1.** Effect of combination of anti-CD20 antibodies and cancer chemotherapeutics in SU-DHL-4 cells. **Table S2.** Effect of combination of anti-CD20 antibodies and cancer chemotherapeutics in RC-K8 cells.
