# Supplementary material for: Novel humanized anti-CD20 antibody BM-ca binds to a unique epitope and exerts stronger cellular activity than others
Source: Cancer Med. 2013 Feb 20;2(2):130–43. doi: 10.1002/cam4.60 (PMC3639652; doi:10.1002/cam4.60)
Supplement: Supplementary file 1 — Figure S1. Combination effects of anti-CD20 antibodies with prednisolone in the anti-cell proliferation assay. Effects of prednisolone at various concentrations were examined in the presence of 0 (•), 0.1 (○), 1 (▴), and 10 μg/mL (▵) of BM-ca (A and D), rituximab (B and E), or ofatumumab (C and F) in SU-DHL-4 (A–C) and RC-K8 (D–F) cells. Each point represents the mean ± SD (n = 4). Figure S2. Combination effects of anti-CD20 antibodies with vincristine in the anti-cell proliferation assay. Effects of vincristine at various concentrations were examined in the presence of 0 (•), 0.1 (○), 1 (▴), and 10 μg/mL (▵) of BM-ca (A and D), rituximab (B and E), or ofatumumab (C and F) in SU-DHL-4 (A–C) and RC-K8 (D–F) cells. Each point represents the mean ± SD (n = 4). Figure S3. Combination effects of anti-CD20 antibodies with hydroxydaunorubicin in the anti-cell proliferation assay. Effects of hydroxydaunorubicin at various concentrations were examined in the presence of 0 (•), 0.1 (○), 1 (▴), and 10 μg/mL (▵) of BM-ca (A and D), rituximab (B and E), or ofatumumab (C and F) in SU-DHL-4 (A–C) and RC-K8 (D–F) cells. Each point represents the mean ± SD (n = 4). Figure S4. Combination effects of anti-CD20 antibodies with cisplatin in the anti-cell proliferation assay. Effects of cisplatin at various concentrations were examined in the presence of 0 (•), 0.1 (○), 1 (▴), and 10 μg/mL (▵) of BM-ca (A and D), rituximab (B and E), or ofatumumab (C and F) in SU-DHL-4 (A–C) and RC-K8 (D–F) cells. Each point represents the mean ± SD (n = 4). Figure S5. Typical representations of gating of lymphocytes (A), histogram without antibody (B), that with BM-ca-FITC (C), and that with rituximab-FITC (D), in flow cytometry analysis of peripheral blood of monkeys (rhesus monkey; animal no. 1). M1 and M2 are negative and positive populations, respectively. Figure S6. Sequences of cDNAs encoding two different types of CD20 molecules in cynomolgus monkeys. In the a.a. 160 = Leu molecule, the nucleotid [file cam40002-0130-sd1.pdf]

Table S1. Effect of combination of anti-CD20 antibodies and cancer chemotherapeutics in SU-DHL-4 cells

Anti-CD20 antibody (μg/mL)		Cancer chemotherapeutics							
		Prednisolone		Vincritine		Hydroxydaunorubicin		Cisplatin	
		IC60 (μM)	inhibition by Ab only (%)	IC60 (nM)	inhibition by Ab only (%)	IC60 (nM)	inhibition by Ab only (%)	IC60 (μM)	inhibition by Ab only (%)
BM-ca	0	45	0.0 ± 2.1	1.5	0.0 ± 2.5	14	0.0 ± 1.7	>100	0.0 ± 4.9
	0.1	0.12	36 ± 1.3	0.66	32 ± 7.0	6.8	33 ± 1.4	60	31 ± 1.1
	1	0.090	43 ± 4.2	0.53	47 ± 9.3	6.6	31 ± 2.8	53	36 ± 2.3
	10	0.074	54 ± 2.1	0.53	42 ± 0.9	6.0	36 ± 1.8	48	40 ± 3.3
Rituximab	0	49	0.0 ± 3.1	1.6	0.0 ± 3.7	17	0.0 ± 2.5	>100	0.0 ± 5.7
	0.1	2.0	26 ± 2.9	0.95	24 ± 4.3	9.0	26 ± 3.7	77	23 ± 0.8
	1	0.074	47 ± 4.7	0.72	34 ± 5.3	6.3	42 ± 1.2	43	40 ± 1.6
	10	<0.032	54 ± 2.0	0.56	43 ± 5.1	4.3	52 ± 1.1	25	50 ± 3.1
Ofatumumab	0	56	0.0 ± 3.1	1.8	0.0 ± 3.0	19	0.0 ± 0.6	97	0.0 ± 2.7
	0.1	32	7.5 ± 2.9	1.5	9.1 ± 4.5	16	13 ± 1.0	84	16 ± 1.0
	1	2.0	20 ± 4.6	1.4	19 ± 6.7	12	29 ± 6.1	53	32 ± 2.1
	10	0.15	37 ± 5.9	0.81	31 ± 4.2	9.3	40 ± 2.4	30	47 ± 6.5

Table S2. Effect of combination of anti-CD20 antibodies and cancer chemotherapeutics in RC-K8 cells

Anti-CD20 antibody (μg/mL)		Cancer chemotherapeutics							
		Prednisolone		Vincritine		Hydroxydaunorubicin		Cisplatin	
		IC60 (μM)	inhibition by Ab only (%)	IC60 (nM)	inhibition by Ab only (%)	IC60 (nM)	inhibition by Ab only (%)	IC60 (μM)	inhibition by Ab only (%)
BM-ca	0	51	0.0 ± 2.3	16	0.0 ± 5.8	17	0.0 ± 3.1	>100	0.0 ± 1.7
	0.1	0.53	34 ± 3.7	2.7	40 ± 4.4	6.0	32 ± 5.6	>100	36 ± 2.9
	1	<0.032	56 ± 3.1	1.8	53 ± 5.0	5.7	43 ± 9.0	>100	32 ± 4.4
	10	23	57 ± 12	0.54	49 ± 3.2	4.6	55 ± 4.9	>100	36 ± 2.9
Rituximab	0	60	0.0 ± 3.8	8.6	0.0 ± 6.0	22	0.0 ± 1.6	>100	0.0 ± 1.4
	0.1	49	8.4 ± 2.3	3.5	19 ± 1.7	19	0.8 ± 3.5	>100	8.5 ± 3.8
	1	49	7.0 ± 2.3	3.3	22 ± 3.5	19	9.1 ± 3.7	>100	15 ± 4.3
	10	45	12 ± 1.3	1.3	28 ± 6.1	17	14 ± 3.3	>100	17 ± 7.6
Ofatumumab	0	57	0.0 ± 2.9	18	0.0 ± 1.7	29	0.0 ± 4.8	>100	0.0 ± 1.0
	0.1	55	1.0 ± 2.4	14	3.8 ± 8.0	25	0.8 ± 5.5	>100	7.6 ± 3.7
	1	54	3.0 ± 5.3	12	3.0 ± 7.7	28	-1.9 ± 5.4	>100	13 ± 9.2
	10	53	2.1 ± 1.9	6.2	4.3 ± 1.2	26	3.2 ± 3.8	74	17 ± 2.5

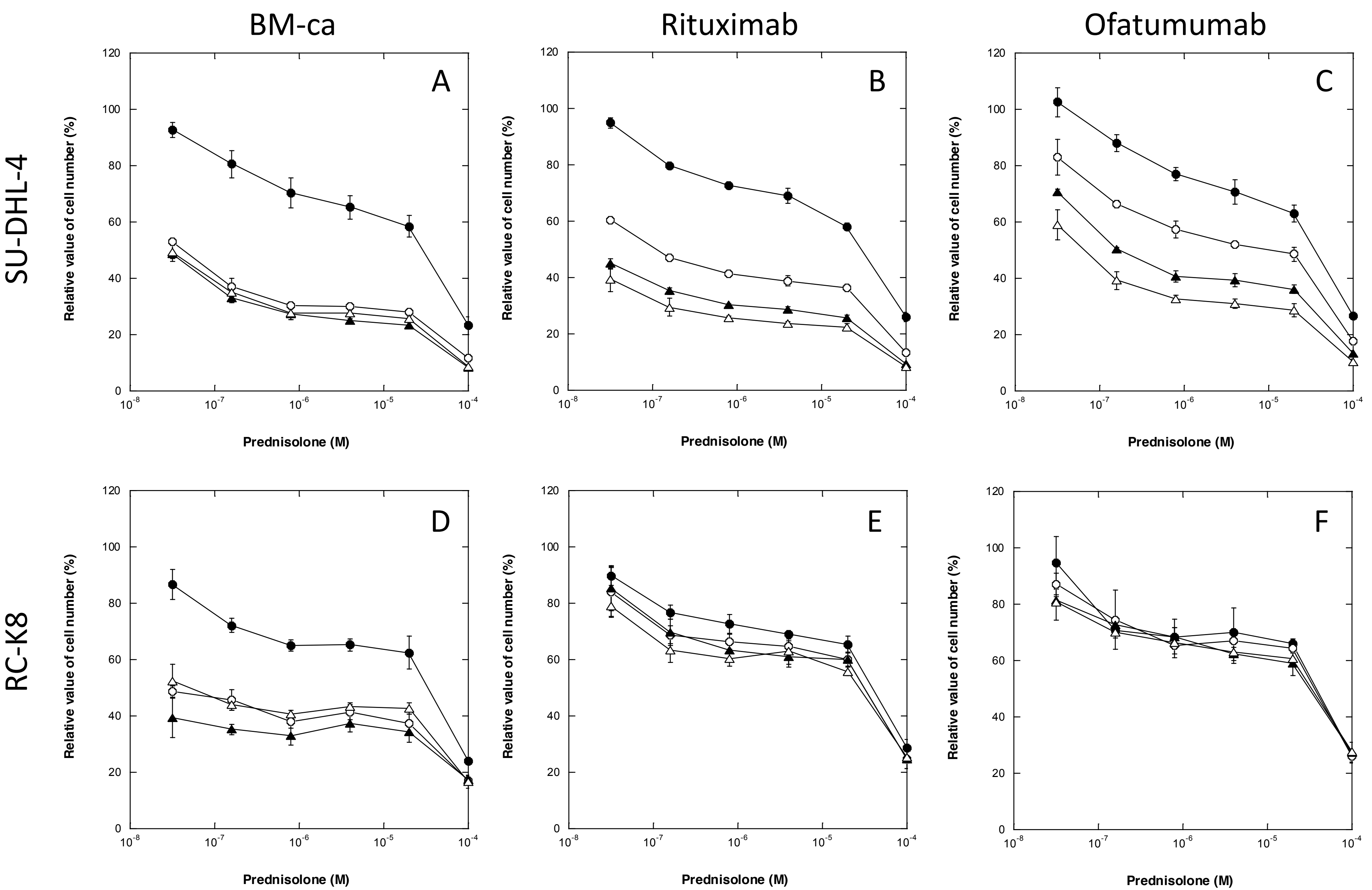

Fig.S1. Combination effects of anti-CD20 antibodies with prednisolone in the anti-cell proliferation assay. Effects of prednisolone at various concentrations were examined in the presence of 0 (●), 0.1 (○), 1 (▲), and 10 μg/mL (Δ) of BM-ca (A and D), rituximab (B and E) or ofatumumab (C and F) in SU-DHL-4 (A, B, and C) and RC-K8 (D, E, and F) cells. Each point represents the mean ± S.D. (n = 4).

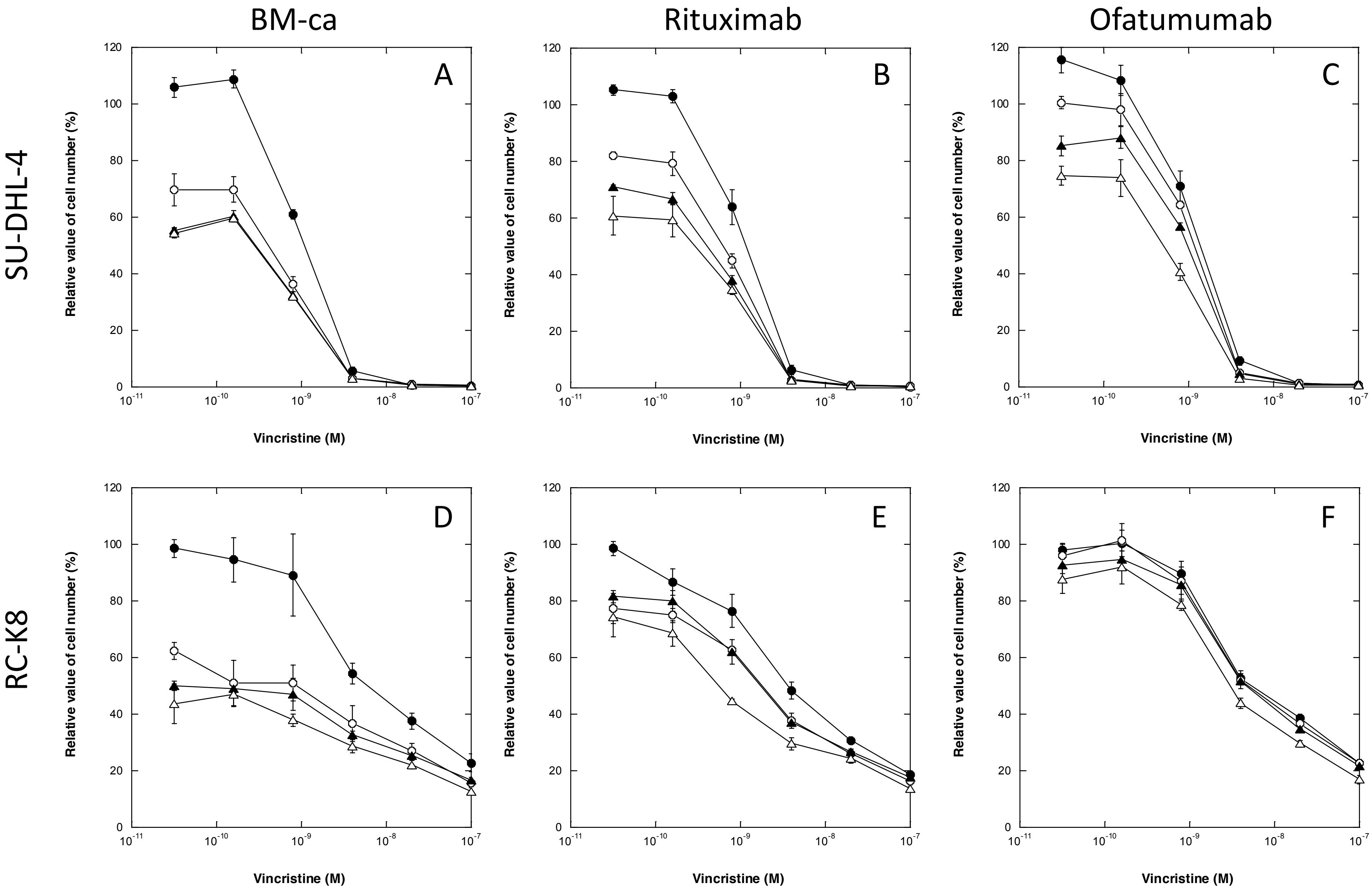

Fig.S2. Combination effects of anti-CD20 antibodies with vincristine in the anti-cell proliferation assay. Effects of vincristine at various concentrations were examined in the presence of 0 (●), 0.1 (○), 1 (▲), and 10 μg/mL (Δ) of BM-ca (A and D), rituximab (B and E) or ofatumumab (C and F) in SU-DHL-4 (A, B, and C) and RC-K8 (D, E, and F) cells. Each point represents the mean ± S.D. (n = 4).

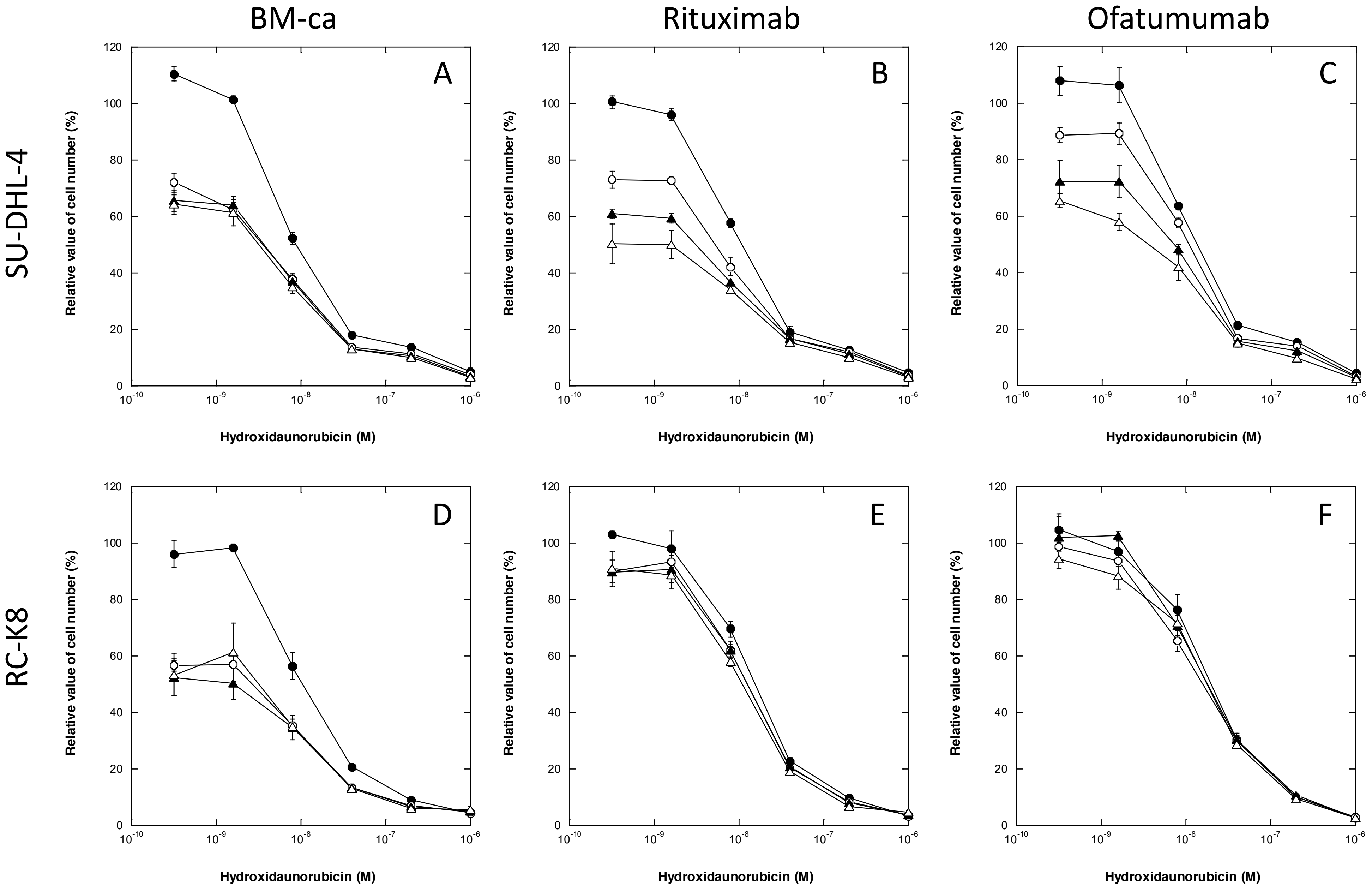

Fig.S3. Combination effects of anti-CD20 antibodies with hydroxudaunorubicin in the anti-cell proliferation assay. Effects of hydroxudaunorubicin at various concentrations were examined in the presence of 0 (●), 0.1 (○), 1 (▲), and 10 μg/mL (Δ) of BM-ca (A and D), rituximab (B and E) or ofatumumab (C and F) in SU-DHL-4 (A, B, and C) and RC-K8 (D, E, and F) cells. Each point represents the mean ± S.D. (n = 4).

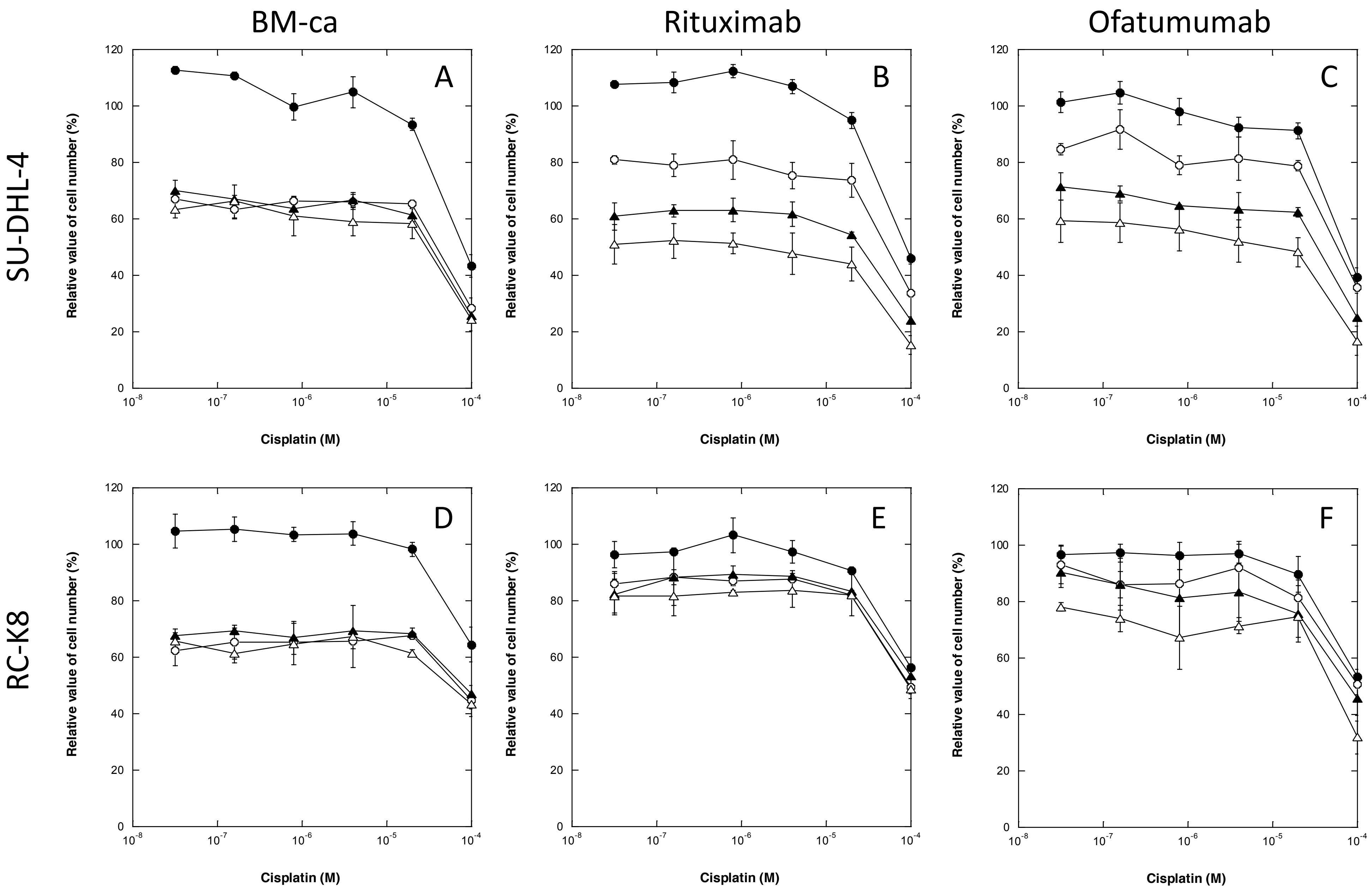

Fig.S4. Combination effects of anti-CD20 antibodies with cisplatin in the anti-cell proliferation assay. Effects of cisplatin at various concentrations were examined in the presence of 0 (●), 0.1 (○), 1 (▲), and 10 μg/mL (Δ) of BM-ca (A and D), rituximab (B and E) or ofatumumab (C and F) in SU-DHL-4 (A, B, and C) and RC-K8 (D, E, and F) cells. Each point represents the mean ± S.D. (n = 4).

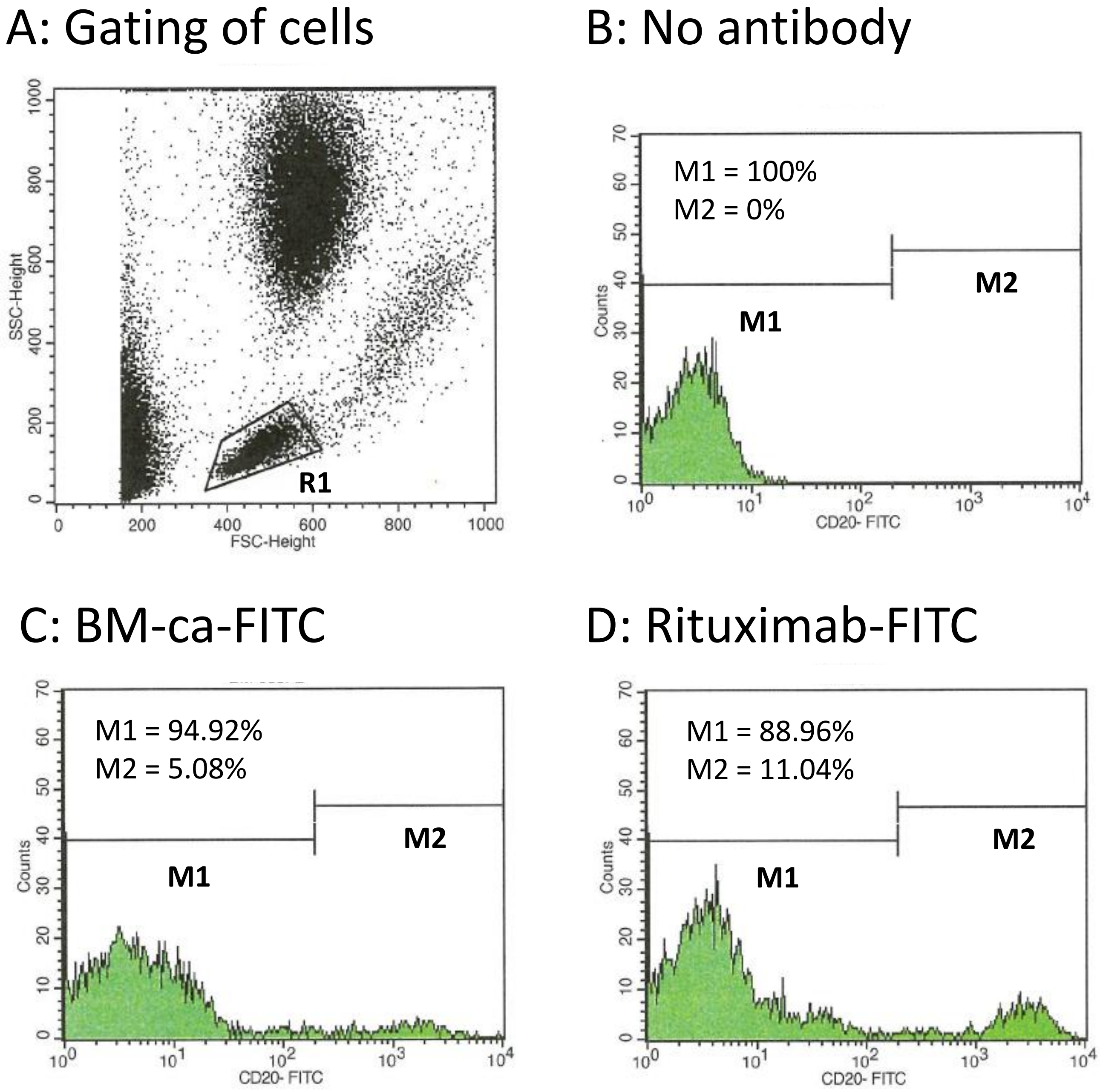

Fig.S5. Typical representations of gating of lymphocytes (A), histogram without antibody (B), that with BM-ca-FITC (C), and that with rituximab-FITC (D), in flow cytometry analysis of peripheral blood of monkeys (rhesus monkey; animal No.1). M1 and M2 are negative and positive populations, respectively.

```
cynomolgus_CD20_AA160Leu    1 ATGACAACACCCAGAAATTCAGTAAATGGGACTTTCCCAGCAGAGCCAATGAAAGGCCCTATTGCTATGCAACCTGGTC
cynomolgus_CD20_AA160Pro    1 ATGACAACACCCAGAAATTCAGTAAATGGGACTTTCCCAGCAGAGCCAATGAAAGGCCCTATTGCTATGCAACCTGGTC
consensus                   1 *******************************************************************************

cynomolgus_CD20_AA160Leu   96 GAGGATGTCTTCACTGGTGGGTCCCACGCAAAGCTTCTTCATGAGGGAATCTAAGGCTTTGGGGGCTGTCCAGATTATG
cynomolgus_CD20_AA160Pro   96 GAGGATGTCTTCACTGGTGGGTCCCACGCAAAGCTTCTTCATGAGGGAATCTAAGGCTTTGGGGGCTGTCCAGATTATG
consensus                  96 *******************************************************************************

cynomolgus_CD20_AA160Leu  191 TTGCCCTGGGGGGTCTTCTGATGATCCCAGCAGGGATCTATGCACCCATCTGTGTGACTGTGTGGTACCCTCTGTGGGG
cynomolgus_CD20_AA160Pro  191 TTGCCCTGGGGGGTCTTCTGATGATCCCAGCAGGGATCTATGCACCCATCTGTGTGACTGTGTGGTACCCTCTGTGGGG
consensus                 191 *******************************************************************************

cynomolgus_CD20_AA160Leu  286 ATTTCCGGATCACTGCTGGCAGCAACGGAGAAAAACTCCAGGAAGTGTTTGGTCAAAGGAAAAATGATAATGAATTCAT
cynomolgus_CD20_AA160Pro  286 ATTTCCGGATCACTGCTGGCAGCAACGGAGAAAAACTCCAGGAAGTGTTTGGTCAAAGGAAAAATGATAATGAATTCAT
consensus                 286 *******************************************************************************

cynomolgus_CD20_AA160Leu  381 CATTTCTGGAATGATTCTTTCAATCATGGACATACTTAATATTAAAATTTCCCATTTTTTAAAAATGGAGAGTCTGAAT
cynomolgus_CD20_AA160Pro  381 CATTTCTGGAATGATTCTTTCAATCATGGACATACTTAATATTAAAATTTCCCATTTTTTAAAAATGGAGAGTCTGAAT
consensus                 381 *******************************************************************************

cynomolgus_CD20_AA160Leu  476 CACTATATATTAACATATACAACTGTGAACCAGCTAATCCCTCTGAGAAAAACTCTCCATCTACTCAATACTGTTACAG
cynomolgus_CD20_AA160Pro  476 CACCATATATTAACATATACAACTGTGAACCAGCTAATCCCTCTGAGAAAAACTCTCCATCTACTCAATACTGTTACAG
consensus                 476 *** ***************************************************************************

cynomolgus_CD20_AA160Leu  571 CTGGGCATTTTGTCAGTGATGCTGATCTTTGCCTTCTTCCAGGAACTTGTAATAGCTGGCATCGTTGAGAATGAATGGA
cynomolgus_CD20_AA160Pro  571 CTGGGCATTTTGTCAGTGATGCTGATCTTTGCCTTCTTCCAGGAACTTGTAATAGCTGGCATCGTTGAGAATGAATGGA
consensus                 571 *******************************************************************************

cynomolgus_CD20_AA160Leu  666 ACCCAAATCTAGCGTAGTTCTCCTGTCAGCTGAAGAAAAAAAAGAACAAGTCATTGAAATAAAAGAAGAAGTGGTTGGG
cynomolgus_CD20_AA160Pro  666 ACCCAAATCTAGCGTAGTTCTCCTGTCAGCTGAAGAAAAAAAAGAACAAGTCATTGAAATAAAAGAAGAAGTGGTTGGG
consensus                 666 *******************************************************************************

cynomolgus_CD20_AA160Leu  761 CCCAACCAAAGAATGAAGAAGACATTGAAATTATTCCAATCCAAGAAGAGGAAGAAGAAGAAACAGAGACAAACTTTCC
cynomolgus_CD20_AA160Pro  761 CCCAACCAAAGAATGAAGAAGACATTGAAATTATTCCAATCCAAGAAGAGGAAGAAGAAGAAACAGAGACAAACTTTCC
consensus                 761 *******************************************************************************

cynomolgus_CD20_AA160Leu  856 CAGGAATCTTCACCAATAGAAAATGACAGCTCTCCTTAA
cynomolgus_CD20_AA160Pro  856 CAGGAATCTTCACCAATAGAAAATGACAGCTCTCCTTAA
consensus                 856 ***************************************
```

Fig.S6. Sequences of cDNAs encoding 2 different types of CD20 molecules in cynomolgus monkeys. In the a.a.160 = Leu molecule, the nucleotide at position 479 is T; whereas in the a.a. 160 = Pro molecule, it is C.

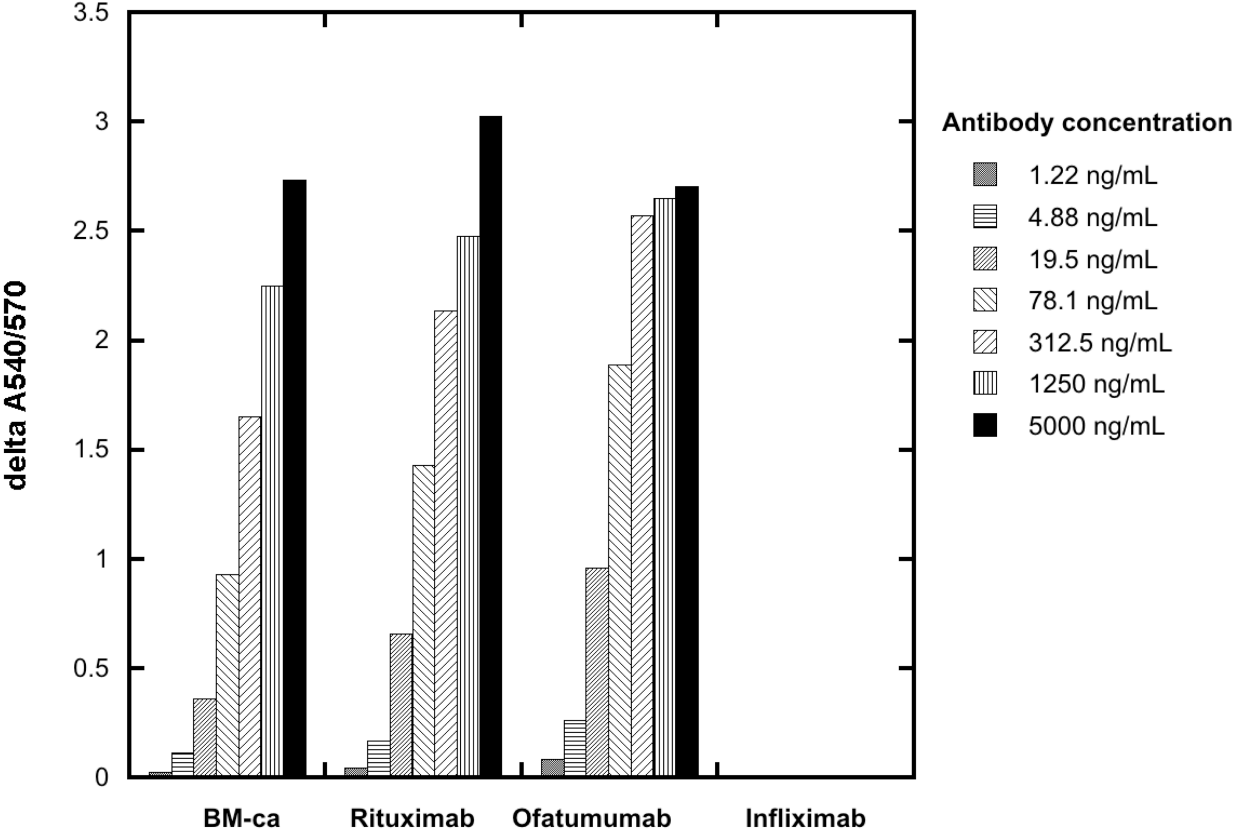

Fig.S7. Reactivity of BM-ca, rituximab, ofatumumab, and infliximab with CD20 molecule expressed on the surface of CHO cells in the ELISA assay under the same assay conditions as in Fig.8.
